# Comparative Evaluation of Antimicrobial and Smear Layer Removal Efficacy of *Mangifera indica* Kernel Extract as Root Canal Irrigant in Primary Molar: An In Vitro Study

**DOI:** 10.1155/2024/5513504

**Published:** 2024-02-07

**Authors:** S. Haripriya, Jamaluddin Mohammed Farzan, Parisa Nourouzi Baghkomeh, Sivakumar Nuvvula

**Affiliations:** ^1^Department of Pediatric and Preventive Dentistry, Faculty of Dentistry, Meenakshi Academy of Higher Education and Research, Meenakshi Ammal Dental College and Hospital, Chennai 600095, Tamil Nadu, India; ^2^Department of Pediatric and Preventive Dentistry, Narayana Dental College and Hospital, Nellore-524003, Andhra Pradesh, India

## Abstract

**Background:**

Endodontic therapy aims to disinfect the entire root canal system. Extracts from the kernel of *Mangifera indica* has the potential to be a novel root canal irrigant that has yet to be studied. Hence, the present study evaluated the antimicrobial, and smear layer removal efficacy of the *M. indica* kernel extract as a root canal irrigant in primary molars.

**Materials and Methods:**

Methanolic extract of *M. indica* was prepared using the standard method. The antimicrobial efficacy of *M. indica* kernel extract was determined by agar diffusion method with 3% sodium hypochlorite and distilled water as controls, and the smear layer removal efficacy was assessed under the SEM after processing the root samples with different concentrations of *M. indica* kernel extract with 17% EDTA and distilled water as positive and negative controls, respectively.

**Results:**

A statistically significant antimicrobial efficacy was observed with the largest mean zone of inhibition recorded with 50 *μ*l of *M. indica* kernel extract at 24 hr of incubation period, when compared to sodium hypochlorite as a root canal irrigant against *Enterococcus faecalis* using agar diffusion method at MIC value of 0.625 mg/ml. The smear layer removal efficacy of the *M. indica* kernel extract was not satisfactory, when compared with EDTA as a root canal irrigant in primary molars and observed under SEM. In contrast, a complete smear layer removal was observed with 17% EDTA solution.

**Conclusion:**

*M. indica* kernel extract has an enhanced antimicrobial efficacy but poor smear layer removal efficacy when used as a root canal irrigant.

## 1. Introduction

Bacteria are the main etiologic factors in developing primary endodontic infections, which occur in untreated root canals, and secondary endodontic infection, which occurs due to the failure of the previous endodontic treatment [1, 2]. *Enterococcus faecalis* is an anaerobic gram-positive coccus, a common microbe in the human oral cavity and gastrointestinal system. It has shown excellent adaptation to habitats with rich nutrients, low oxygen levels, and complicated ecology. Moreover, endodontic infections have been linked to Enterococci. *E. faecalis* is the most prevalent *Enterococci* species found in root canals. The bacterium infected root canals is nonfastidious and therapy-resistant [3]. Endodontic therapy aims to disinfect the entire root canal system and prevent reinfection during and after treatment. This goal is pursued by chemo-mechanical debridement, combining the mechanical systems with the irrigating solutions [4]. For teeth with intricate internal architecture, such as fins or other anomalies that instruments might overlook, chemical debridement is essential [5]. Saline is commonly used as an endodontic irrigant as it is safe and nontoxic to the periapical tissues, but it does not have an antibacterial and smear layer removal efficacy; hence, it is used in conjunction with other irrigation solutions, whereas sodium hypochlorite (NaOCl) has an antibacterial effect, can dissolve the tissues, quickly diffuse on the walls of the dentin, is easily accessible and economical but it is also toxic when it is extruded from the apex thereby accelerates the root resorption [6]. Herbs, spices, and plant-derived chemicals have specific antibacterial, antifungal, and biocompatible properties, which have led to the discovery of herbal root canal disinfectants [7]. The benefits of herbal root canal irrigants include their safety, accessibility, affordability, and lack of bacterial resistance, and they can be potential agents when combined with other conventional irrigants for successful root canal therapy [8]. Ethylenediaminetetraacetic acid (EDTA) is a polyamine carboxylic acid that acts as a hexadentate ligand and chelating agent for effective smear layer removal. It reacts with the calcium ions of the dentin and forms soluble calcium chelates. It has been reported that EDTA decalcified dentin to a depth of 20–30 *μ*m in 5 min [9].

Herbal medicinal plants efficiently treat infectious diseases while at the same time minimizing some of the side effects commonly associated with synthetic antimicrobials. Mango has provided humanity with a lot of such medicinal benefits [10]. It has been an essential herb in the Ayurvedic and indigenous medical systems for over 4,000 years. Mangiferin in *Mangifera indica* is a polyphenolic antioxidant and a glucosyl xanthone resulting in antioxidant and antilipid peroxidation activities. Due to its antibacterial and antifungal properties, various parts of *M. indica* are used to prepare dentifrices, antiseptics, and astringents [11]. The kernel, however, might be used for other purposes outside food if it is given the right treatment and research. Mango seed kernel fat was shown to be healthy and nontoxic, allowing it to be substituted for any solid fat without having negative consequences, according to nutritional and toxicological investigations done on the mango seed kernel [12]. Extracts from the kernel of *M. indica* has the potential to be a novel root canal irrigant with the properties mentioned above, and its efficacy has not been studied as an irrigant to disinfect the root canal system. The efficacy of a root canal irrigant is usually studied using its antimicrobial and smear layer removal properties. Hence, the present study evaluated the antimicrobial and smear layer removal efficacy of the *M. indica* kernel extract as a root canal irrigant in primary molars. The objectives of the study were to evaluate the antimicrobial efficacy of the *M. indica* kernel extract in comparison with 3% NaOCl used as a root canal irrigant against *E. faecalis* using the agar diffusion method, and to evaluate the smear layer removal efficacy of the *M. indica* kernel extract in comparison with 17% EDTA as a root canal irrigant in primary molars using scanning electron microscope (SEM).

## 2. Materials and Methods

A methanolic extract of *M. indica* was prepared by mixing 50 g of commercially available mango kernel powder with 100 ml of methanol by diffusion and filtration. The minimum inhibitory concentration (MIC) of this extract was determined by serial dilution method. In this step wise dilution method, the mango kernel extract along with the broth is diluted with the same dilution factor at each tube till the 10^th^ tube as we followed the tenfold serial dilution method. After the serial dilution, the stock solution, BHI broth and the organism *E. faecalis* were added to the tubes for the reaction. Then, the color change is observed at the 5^th^ tube at the dilution of 0.625 mg/ml. Followed by the determination of the antimicrobial efficacy by agar diffusion method, the smear layer removal efficacy was assessed under the SEM. Ten brain heart infusion (BHI) agar plates (HI MEDIA, Mumbai, India—M210-500G) were prepared, and strains of *E. faecalis* (ATCC-29212- KWIK-STIK™, Microbiologics, Minnesota, USA) were grown in 2 ml of BHI broth for 24 hr at 37°C, and this *E. faecalis* inoculum was streaked on the prepared BHI agar plates using a sterile cotton swab followed by incubation for 24 hr at 37°C. In each of the inoculated BHI agar plates, four wells were prepared measuring about 6 mm in diameter; the first two wells were loaded with the *M. indica* kernel extract based on the MIC values obtained; the third and the fourth wells were loaded with 3% of NaOCl (prime dental products, Khaler, India; positive control) and distilled water (negative control), respectively and were incubated for 24 hr at 37°C. The zone of inhibition around each well was then measured, and the data collected were tabulated and statistically analyzed using SPSS (IBM SPSS Statistics for Windows, Version 26.0, Armonk, NY: IBM Corp. Released 2019).

The smear layer removal efficacy was assessed using SEM by measuring the pore size of the dentinal tubules in the root canals of primary molars after irrigating with the *M. indica* kernel extract. Fifteen extracted primary molars indicated for extraction for orthodontic reasons were collected, and the roots of minimum 9 mm in length were sectioned at the cemento enamel junction (CEJ) using a diamond disc. A total of 21 intact roots were selected for the study, for which working length and biomechanical preparation were done using a K-file, and saline irrigation was done with 10 ml syringe with 21 gauge needle (DispoVan 10 ml syringe—Hindustan Syringes & Medical Devices Ltd., Faridabad, Haryana, India). The prepared roots were randomly assigned to three irrigant groups: *M. indica* kernel extract solution, 17% EDTA solution (DESMEAR EDTA 17% solution 150 ml bottle), and Sterile water. The irrigation was done with 10 ml of the respective irrigants for 2 min. The irrigated roots were then cleaved longitudinally into two parts using a surgical chisel and mallet to expose the entire canal extension. Torabinejad's criteria [13] among the 21 prepared roots, only the root surface which was properly cleaved with chisel and mallet were selected for the study and the rest of them were discarded. Seven sectioned root samples were dried in a hot air oven at 50°C for about 5 hr, coated with palladium, and subjected to SEM examination. Micrographs were obtained at 1,000x, 2,000x, and 13,000x magnifications at the middle third of the roots to visualize the smear layer. The obtained micrographs were qualitatively analyzed, and scored as Score 0, no smear layer, all dentinal tubules open, and no erosion of tubules; Score 1, no smear layer, all dentinal tubules open, and erosion of tubules; Score 2, minimum smear layer >50% dentinal tubules visible; Score 3, moderate smear layer, <50% of dentinal tubules open; and Score 4, heavy smear layer, outline of dentinal tubules obliterated [14]. The collected data were tabulated and statistically analyzed using SPSS (IBM SPSS Statistics for Windows, Version 26.0, Armonk, NY: IBM Corp. Released 2019).

## 3. Results

The serial dilution method showed that 0.625 mg/ml was the MIC value for the prepared *M. indica* kernel extract; hence, the first two wells were loaded with 50 and 40 *μ*l of the 0.625 mg/ml of *M. indica* kernel extract. The measured values of the zone of inhibition were analyzed for normality using Kolmogorov–Smirnov and Shapiro–Wilks tests, which revealed that the obtained values do not follow a normal distribution. Therefore, the Kruskal–Wallis test was used to analyze the data, followed by the Bonferroni test for pairwise comparison of groups.  Table 1 shows the mean ± SD of the zone of inhibition of *E. faecalis* for all four wells. The values observed in a well containing 50 *μ*l of 0.625 mg/ml of *M. indica* kernel extract showed the maximum antimicrobial efficacy, followed by a well containing 40 *μ*l of 0.625 mg/ml of *M. indica* kernel extract; the well containing NaOCl showed the least efficacy, followed by no antimicrobial efficacy seen in well containing saline. Upon pairwise comparisons of the materials, the results showed that both 50 and 40 *μ*l of 0.625 mg/ml of *M. indica* kernel extract exhibited the maximum antibacterial activity when compared to saline and NaOCl, which was statistically significant (*p* < 0.001).

**Figure 1:**
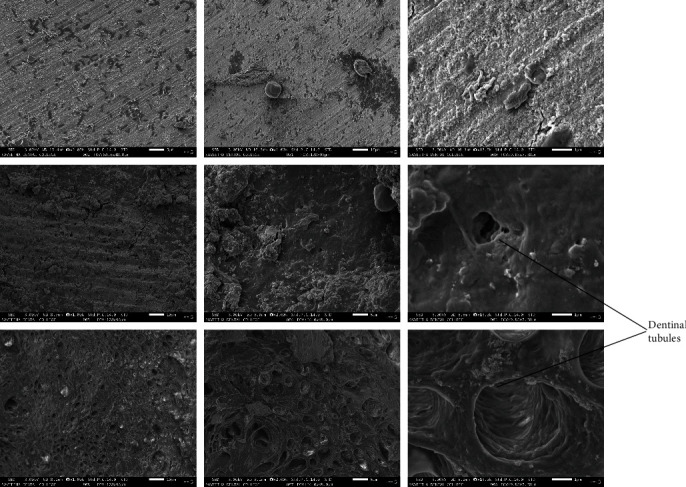
SEM micrographs of root sections irrigated with *M. indica* kernel extract.

**Figure 2:**
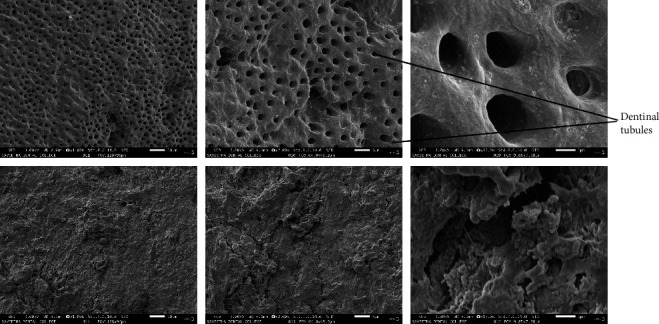
SEM micrographs of root sections irrigated with 17% EDTA.

**Figure 3:**
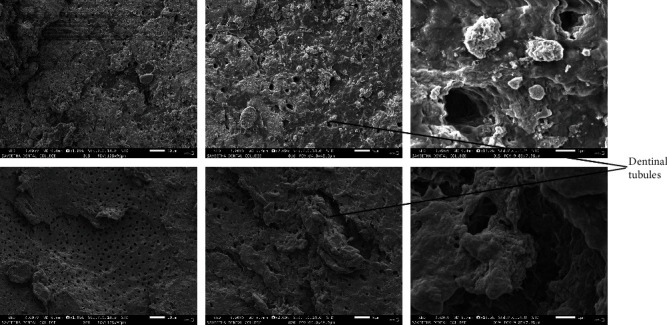
SEM micrographs of root sections irrigated with sterile water.

The SEM examination revealed that only seven root sections were efficiently coated with palladium for proper visualization; three from the *M. indica* kernel extract group (Figure 1), two from the 17% EDTA group (Figure 2), and two from the sterile water group (Figure 3). The smear layer scores from the micrographs were analyzed for normality using Kolmogorov–Smirnov and Shapiro–Wilks tests, which revealed that the obtained values do not follow a normal distribution. Therefore, the Kruskal–Wallis test was used to analyze the data and to compare the scores between the groups.  Table 2 shows the comparison of the mean ± SD scores for the smear layer of the root canal surfaces seen under SEM, which showed that the highest smear layer removal efficacy against *E. faecalis* was observed in the 17% EDTA group, followed by *M. indica* kernel extract group, which was not statistically significant. The sterile water group did not exhibit any smear layer removal.

## 4. Discussion

Studies have reported that *M. indica* kernel extract showed good antimicrobial activity when used as a methanolic preparation; hence, a similar preparation was used in the present study [15, 16]. It was considered not to exceed the acute toxic dose of *M. indica* kernel extract [17]; hence, 50 and 40 *μ*l of *M. indica* kernel extract were used to test the herbal agents. Among the test agents compared against *E. faecalis*, the highest zone of inhibition was observed for *M. indica* kernel extract when compared to NaOCl, which was statistically significant. The available scientific evidence suggests that *M. indica* possesses antimicrobial properties against gram-positive and gram-negative bacteria [10]. *M. indica* kernel extract showed more significant antimicrobial activity, when compared to 5.25% NaOCl and 2% chlorhexidine against *E. faecalis* [18]. The *M. indica* kernel extract contains coumarins, terpenes, tannins, and flavonoids, which may be responsible for its high antibacterial activity [19]. Also, the high concentration of major phenolic compounds such as hesperidin is responsible for scavenging free radicals, which may have encouraged its antibacterial properties [20, 21]. Previous studies have shown that various herbal agents, when used as an irrigating solution compared to NaOCl, have effectively removed the smear layer from the root canal system [22, 23]. The samples in this study were studied at 1,000x, 2,000x, and 13,000x magnifications and scored according to the scoring system given by Rome et al. [14] in 1985 as follows:

Score 0, no smear layer, all dentinal tubules open, and no erosion of tubules.

Score 1, no smear layer, all dentinal tubules open, and erosion of tubules.

Score 2, minimum smear layer >50% dentinal tubules visible.

Score 3, moderate smear layer, <50% of dentinal tubules open.

Score 4, heavy smear layer, outline of dentinal tubules obliterated

This is the first study to investigate the smear layer removal efficacy of *M. indica* kernel extract, and the results of this study showed that the smear layer removal efficacy of the gold standard agent EDTA was better than that of the *M. indica* kernel extract. To our knowledge, no previous studies assessed the smear layer removal efficacy of *M. indica* kernel extract.

Many other studies on various other herbal agents used as an endodontic irrigation solution were found to be effective in removing the smear layer. Mukherjee et al. [24] concluded that Triphala is as effective as EDTA in removing the smear layer from root canals during endodontic procedures. Sharma and Dhawan [22] examined the smear layer removal performance of green tea extract, tulsi leaf extract, and neem leaf extract compared with sodium hypochlorite. The neem extract showed the highest smear layer removal efficacy in treated canals.

It may be understood from the results of this study that *M. indica* kernel extract may be a potent antimicrobial solution but not a good smear layer removal agent. Hence, a combination of *M. indica* kernel extract with other herbal agents with a good smear layer removal efficacy may be investigated as an endodontic irrigant. The limitation of this study is that this is an in vitro study, and the results of this study may not be directly extrapolated for clinical situations as the choice of an irrigating solution would depend on each patient's clinical scenario. Hence, further clinical studies are required to establish the antimicrobial efficacy of *M. indica* kernel extract. Further, in vitro study with a larger sample size is required to confirm the results from this study regarding the smear layer removal efficacy of *M. indica* kernel extract when used as an irrigating solution.

## 5. Conclusions

The following conclusions can be drawn within the limits of this in vitro study. A statistically significant antimicrobial efficacy was observed with the largest mean zone of inhibition recorded with 50 *μ*l of *M. indica* kernel extract at 24 hr of incubation period, when compared to NaOCl as a root canal irrigant against *E. faecalis* using agar diffusion method at MIC value of 0.625 mg/ml. The smear layer removal efficacy of the *M. indica* kernel extract was not satisfactory compared with EDTA as a root canal irrigant in primary molars, when observed under SEM. In contrast, a complete smear layer removal was observed with 17% EDTA solution.

It can be concluded that *M. indica* kernel extract has an enhanced antimicrobial efficacy but poor smear layer removal efficacy, when used as root canal irrigant. The choice of an irrigating solution would depend on each patient's clinical scenario. Since natural therapy has a number of positive characteristics, including antibacterial, antifungal, and biocompatible capabilities along with no cytotoxicity and it does not reduce the microhardness of the root dentin, a variety of herbal and plant derived chemicals have been identified as possible root canal disinfectants. Further clinical studies are required to confirm the findings of the present study.

## Figures and Tables

**Table 1:** Mean ± SD zone of inhibition of *E. faecalis* for the four irrigating solutions.

Well	Irrigant solution	*N*	Mean ± SD^ ^*∗*^Kruskal–Wallis test^	*p*
1	*M. indica* kernel extract (50 *μ*l of 0.625 mg/ml)	10	16.29 ± 2.428	<0.001
2	*M. indica* kernel extract (40 *μ*l of 0.625 mg/ml)	10	14.71 ± 2.514	
3	3% of sodium hypochlorite (positive control)	10	4.78 ± 3.325	
4	Distilled water (negative control)	10	0.00 ± 0.000	

The mean and standard deviation values of the four wells were measured (*N* = 10) using the Kruskal–Wallis test. The mean values observed with 50 *μ*l of 0.625 mg/ml of *M. indica* kernel extract showed the highest antimicrobial efficacy against *E. faecalis* (16.29), followed by 40 *μ*l of 0.625 mg/ml of *M. indica* kernel extract. 50 *μ*l of 3% of sodium hypochlorite showed the least efficacy, and no antimicrobial efficacy was seen with 50 *μ*l of distilled water.

**Table 2:** Intergroup comparison of the scores seen under SEM.

Irrigant solution	*N*	Mean ± SD^ ^*∗*^Kruskal–Wallis test^		
		1,000x	2,000x	13,000x
*M. indica* kernel extract	3	2.00 ± 1.00	3.00 ± 1.00	3.00 ± 1.00
17% EDTA	2	0.50 ± 0.71	0.50 ± 0.71	0.00 ± 0.00
Sterile water	2	4.00 ± 0.00	4.00 ± 0.00	3.50 ± 0.71
*p*		0.080	0.090	0.125

Comparison of the scores for the smear layer of the root canal surfaces seen under SEM. The mean values observed with 17% EDTA solution showed the highest smear layer removal efficacy against *E. faecalis* followed by *M. indica* kernel extract solution, which is not statistically significant. There was no smear layer removal observed with sterile water.

## Data Availability

The data used to support the findings of this study will be available from the corresponding author upon request.

## References

[1] Singh H. (2016). Microbiology of endodontic infections. *Journal of Dental and Oral Health*.

[2] Gajan E. B., Aghazadeh M., Abashov R., Milani A. S., Moosavi Z. (2009). Microbial flora of root canals of pulpally-infected teeth: *Enterococcus faecalis* a prevalent species. *Journal of Dental Research, Dental Clinics, Dental Prospects*.

[3] Alghamdi F., Shakir M. (2020). The influence of enterococcus faecalis as a dental root canal pathogen on endodontic treatment: a systematic review. *Cureus*.

[4] Plotino G., Cortese T., Grande N. M. (2016). New technologies to improve root canal disinfection. *Brazilian Dental Journal*.

[5] Baker N. A., Eleazer P. D., Averbach R. E., Seltzer S. (1975). Scanning electron microscopic study of the efficacy of various irrigating solutions. *Journal of Endodontics*.

[6] Daloğlu M., Güzel K. G. U. (2017). Root canal treatment for deciduous teeth: a review. *Meandros Medical and Dental Journal*.

[7] Wong J., Manoil D., Näsman P., Belibasakis G. N., Neelakantan P. (2021). Microbiological aspects of root canal infections and disinfection strategies: an updated review on the current knowledge and challenges. *Frontiers in Oral Health*.

[8] Sahni A., Chandak M. G. (2015). Herbal usage in root canal irrigation: a review. *International Journal of Dental and Health Sciences*.

[9] Mohammadi Z., Shalavi S., Jafarzadeh H. (2013). Ethylenediaminetetraacetic acid in endodontics. *European Journal of Dentistry*.

[10] Osei-Djarbeng S. N., Kwarteng R. O., Osei-Asante S., Owusu-Dapaah G. (2020). Comparative antimicrobial activities of ethanolic extracts of leaves, seed and stem bark of *Mangifera indica* (Mango). *Journal of Pharmacognosy and Phytochemistry*.

[11] Shah K. A., Patel M. B., Patel R. J., Parmar P. K. (2010). *Mangifera indica* (Mango). *Pharmacognosy Reviews*.

[12] Karunanithi B., Bogeshwaran K., Tripuraneni M., Reddy S. K. (2015). Extraction of mango seed oil from mango kernel. *International Journal of Engineering Research and Development*.

[13] Venkatachalamoorthi V., Shivashankarappa P. G., Adimoulame S., Gurusamy K., Muthukrishnan K., Govindan E. (2023). Effect of passion fruit juice in removal of smear layer in root canal of *Ex Vivo* human teeth: a scanning electron microscopic study. *International Journal of Clinical Pediatric Dentistry*.

[14] Rome W. J., Doran J. E., Walker W. A. (1985). The effectiveness of Gly-Oxide and sodium hypochlorite in preventing smear layer formation. *Journal of Endodontics*.

[15] Raju N. V., Sukumar K., Reddy G. B. (2019). In-vitro studies on antitumor and antimicrobial activities of methanolic kernel extract of *Mangifera indica* L. cultivar banganapalli. *Biomedical & Pharmacology Journal*.

[16] Abdalla A. E. M., Darwish S. M., Ayad E. H. E., El-Hamahmy R. M. (2007). Egyptian mango by-product 2: antioxidant and antimicrobial activities of extract and oil from mango seed kernel. *Food Chemistry*.

[17] Ashalatha M., kumar S., Janadri S., Yoganand S. R. J. B. (2015). Acute toxicity study of seed kernel of *Mangifera indica* linn (SKMI). *International Ayurvedic Medical Journal*.

[18] Viswanath S., Thomas G., Jose S., Madhukiran M. K., Joseph S., Thankachan T. (2020). Comparative evaluation of antimicrobial efficacy of ocimum sanctum, tridax procumbens, and mango kernel extracts with sodium hypochlorite and chlorhexidine as root canal irrigants—an in vitro study. *Indian Journal of Contemporary Dentistry*.

[19] Ahmed I. S., Tohami M. S. E., Almagboul A. Z., Verpoorte R. (2012). Characterization of antimicrobial compounds isolated from *Mangifera indica* L. seed kernel. *University of Africa Journal of Sciences*.

[20] Vaghasiya Y., Chanda S. (2010). Antimicrobial and free radical scavenging activity of different solvent extracts of *Mangifera indica* L. seeds. *Research Journal of Microbiology*.

[21] Abdel-Aty A. M., Salama W. H., Hamed M. B., Fahmy A. S., Mohamed S. A. (2018). Phenolic-antioxidant capacity of mango seed kernels: therapeutic effect against viper venoms. *Revista Brasileira de Farmacognosia*.

[22] Sharma K., Dhawan R. (2021). Efficacy of herbal extracts in the removal of smear layer: an in-vitro study. *International Journal of Scientific Research*.

[23] Sowjanyaa J., Thomas T., Chandana C. S. (2017). Comparative evaluation of the efficacy of smear layer removal by ethylenediaminetetraacetic acid, *Triphala*, and *German chamomile* as irrigants—a scanning electron microscopy study. *Journal of Advanced Pharmacy Education & Research*.

[24] Mukherjee M., Kalita T., Barua P. (2023). Efficacy of smear layer removal of human teeth root canals using herbal and chemical irrigants: an in vitro study. *Cureus*.

